# *Cocos nucifera* (L.) (Arecaceae): A phytochemical and pharmacological review

**DOI:** 10.1590/1414-431X20154773

**Published:** 2015-08-18

**Authors:** E.B.C. Lima, C.N.S. Sousa, L.N. Meneses, N.C. Ximenes, M.A. Santos, G.S. Vasconcelos, N.B.C. Lima, M.C.A. Patrocínio, D. Macedo, S.M.M. Vasconcelos

**Affiliations:** 1 Laboratório de Neuropsicofarmacologia, Departamento de Fisiologia e Farmacologia, Faculdade de Medicina, Universidade Federal do Ceará, Fortaleza, CE, Brasil; 2 Laboratório de Farmacologia, Curso de Medicina, Centro Universitário Christus-Unichristus, Fortaleza, CE, Brasil

**Keywords:** *Cocos nucifera* (L.), Ethnopharmacology, Pharmacological properties, Biological activity, Review

## Abstract

*Cocos nucifera* (L.) (Arecaceae) is commonly called the “coconut tree” and is the most naturally widespread fruit plant on Earth. Throughout history, humans have used medicinal plants therapeutically, and minerals, plants, and animals have traditionally been the main sources of drugs. The constituents of *C. nucifera* have some biological effects, such as antihelminthic, anti-inflammatory, antinociceptive, antioxidant, antifungal, antimicrobial, and antitumor activities. Our objective in the present study was to review the phytochemical profile, pharmacological activities, and toxicology of *C. nucifera* to guide future preclinical and clinical studies using this plant. This systematic review consisted of searches performed using scientific databases such as Scopus, Science Direct, PubMed, SciVerse, and Scientific Electronic Library Online. Some uses of the plant were partially confirmed by previous studies demonstrating analgesic, antiarthritic, antibacterial, antipyretic, antihelminthic, antidiarrheal, and hypoglycemic activities. In addition, other properties such as antihypertensive, anti-inflammatory, antimicrobial, antioxidant, cardioprotective, antiseizure, cytotoxicity, hepatoprotective, vasodilation, nephroprotective, and anti-osteoporosis effects were also reported. Because each part of *C. nucifera* has different constituents, the pharmacological effects of the plant vary according to the part of the plant evaluated.

## Introduction

*Cocos nucifera* (L.) is an important member of the family Arecaceae (palm family) popularly known as coconut, coco, coco-da-bahia, or coconut-of-the-beach (1). The plant is originally from Southeast Asia (Malaysia, Indonesia, and the Philippines) and the islands between the Indian and Pacific Oceans. From that region, the fruit of the coconut palm is believed to have been brought to India and then to East Africa. After the discovery of the Cape of Good Hope, this plant was introduced into West Africa and, from there, dispersed to the American continent and to other tropical regions of the globe (2).

**Figure 1:**
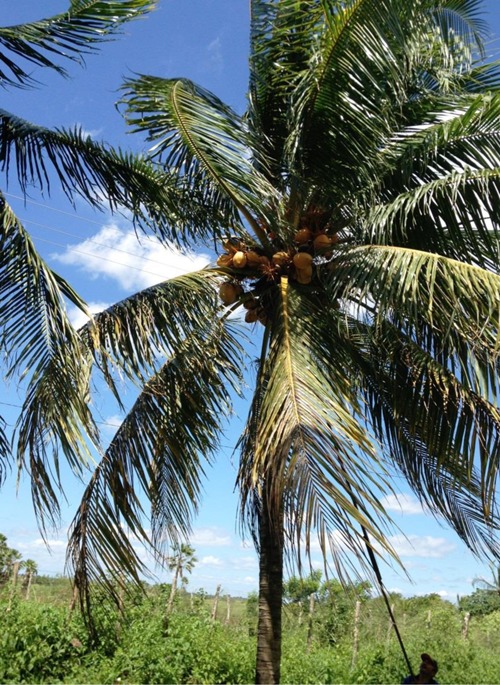
A, *Cocos nucífera* L. (personal collection, agronomist Severo Cortez Lima).

The plant is an arborescent monocotyledonous tree of around 25 m in height (giant coconut) with a dense canopy (Figure 1). The root of the coconut system is fasciculated. The stem is an unbranched type, and at its apex, a tuft of leaves protects a single apical bud. The pinnate leaves are feather-shaped, having a petiole, rachis and leaflets. Under favorable environmental conditions, the giant adult coconut emits 12–14 inflorescence spikes per year, while the adult dwarf coconut can emit 18 spikes in the same period. The axillary inflorescence has globular clusters of female flowers. The plant is monoecious (male and female reproductive organs on the same plant) (3).

The coconut fruit comprises an outer epicarp, a mesocarp, and an inner endocarp. The epicarp, which is the outer skin of the fruit, and the mesocarp, which is heavy, fibrous, and tanned when dry, have many industrial uses. The endocarp is the hard dark core. Inside is a solid white albumen of varied thickness, depending on the age of the fruit, and with an oily pulp consistency and a liquid albumen called coconut water that is thick, sweet, and slightly acidic (3,4). The authors and the synonyms of the plant were confirmed using www.theplantlist.org (Table 1).

**Table:**
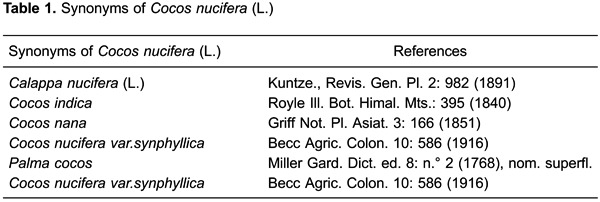

The present review highlights the traditional uses of *C. nucifera*, phytochemical compounds isolated from different parts of the plant, and the biological activity and toxicological studies to date.

## Methods

Articles published in English were searched in the online databases Scopus, Science Direct, PubMed, SciVerse and Scientific Electronic Library Online (SciELO), with no time limits. Search terms included combinations of the following: ‘*Cocos nucifera*’, ‘*C. nucifera* and phytochemical profile’, ‘*C. nucifera* and pharmacological properties’, and ‘*C. nucifera* and toxicology’.

### Traditional uses

All parts of the fruit of the coconut tree can be used. Both the green coconut water and solid albumen ripe fruits are used industrially and in home cooking in many ways (5). Additionally, several parts of the fruit and plant have been used by people in different countries for the treatment of various pathological conditions (Table 2).

**Table:**
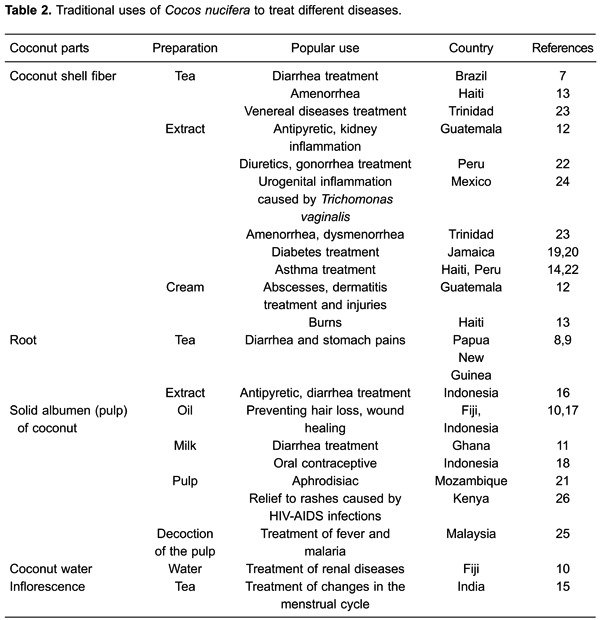

Currently, appreciation of natural coconut water is growing. Industry is using the husk fiber from the pith as raw material for carpets, car seat stuffing, and in agricultural as fertilizers. The hard core is used to make handcrafts. The stalk and leaves of the coconut tree are useful in construction, and sugar, vinegar, and alcohol can be extracted from the inflorescence (6).

In Brazil, extract from the husk fiber of *C. nucifera* is used to treat diarrhea (7). In Papua New Guinea, the leaves and roots of young plants are chewed as treatment for diarrhea and stomachaches (8,9). In Fiji, coconut oil is used to prevent hair loss and coconut water is used to treat renal disease (10). In Ghana, people use coconut milk to treat diarrhea (11). In Guatemala, the husk fiber extract is used as an antipyretic, to reduce renal inflammation, and as a topic ointment for dermatitis, abscesses, and injuries (12). In Haiti, a decoction of the dry pericarp is used for oral treatment of amenorrhea, and the oil is applied as an ointment to burns (13); an aqueous extract from the husk fiber is also used for oral asthma treatment (14). In India, infusions made with the coconut inflorescence are used for the oral treatment of menstrual cycle disorders (15). In Indonesia, the oil is used as a wound ointment, the coconut milk is used as an oral contraceptive, and fever and diarrhea are treated with the root extract (16–18). In Jamaica, the husk fiber extract is used to treat diabetes (19,20). In Mozambique, the fruit is consumed by men as an aphrodisiac (21). Peruvians use the aqueous extract of the fresh coconut fiber orally for asthma, as a diuretic, and for gonorrhea (22). In Trinidad, bark extract is used orally for amenorrhea and dysmenorrhea, and bark tea is used to treat venereal diseases (23). In Mexico, coconut is used to treat various disorders associated with urogenital tract infection by *Trichomonas vaginalis* (24). A decoction of the white flesh of the fruit is used in rural Malaysia to treat fever and malaria (25). In Kenya, the fruit is used to relieve skin rash caused by HIV infection (26).

### Phytochemistry

Phytochemical studies of the coconut fiber (mesocarp) ethanolic extract revealed that the presence of phenols, tannins, leucoanthocyanidins, flavonoids, triterpenes, steroids, and alkaloids (27), while a butanol extract recovered triterpenes, saponins, and condensed tannins (28). Notably, compounds like flavonoids having antioxidant action are widely distributed in edible vegetables, fruits, and many herbs (29–31). Condensed tannins are reported to possess antihelminthic activity by binding to proteins present in the cuticle, oral cavity, esophagus, and cloaca of nematodes, thus intensifying the physical and chemical damage in helminth (32).

The lyophilized extract and fractions, as well as ethyl acetate extracts, from the *C. nucifera* fiber are rich in polyphenols, compounds such as catechins, epicatechins, tannins, and flavonoids (7,33–35).

**Figure 2:**
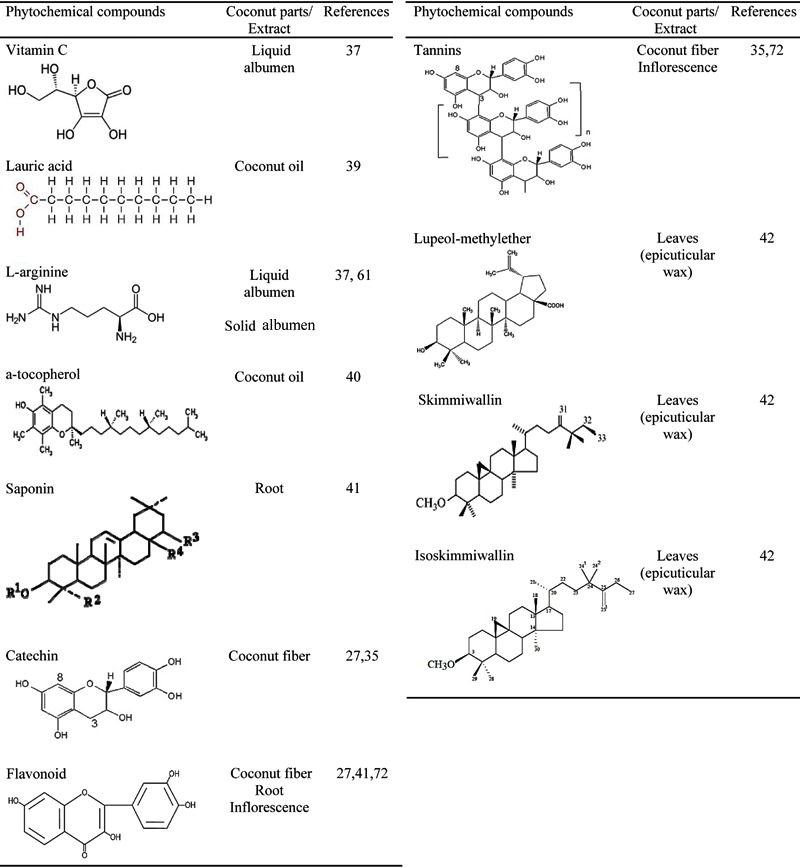
Structures of the main phytoconstituents isolated from *Cocos nucifera* (L.).

The constituents of the liquid albumen were identified as vitamin B, nicotinic acid (B3, 0.64 µg/mL), pantothenic acid (B5, 0.52 µg/mL), biotin (0.02 µg/mL), riboflavin (B2, <0.01 ng/mL), folic acid (0.003 µg/mL), with trace quantities of vitamins B1, B6, and C, pyridoxine, thiamine, folic acid, amino acids, L-arginine, plant hormones (auxin, 1,3-diphenylurea, cytokinin), enzymes (acid phosphatase, catalase, dehydrogenase, diastase, peroxidase, RNA polymerases), and growth-promoting factors (36–38). Furthermore, oil extracted from the solid albumen is primarily lauric acid and alpha tocopherol (39,40). Root phenolic compounds were identified as flavonoids and saponins (41). Other compounds identified in leaf epicuticular wax were lupeol methylether, skimmiwallin, [3b-methoxy-25-ethyl-9,19-cyclolanost-24(241)-ene], and isoskimmiwallin [3b-methoxy-24-ethyl-9,19-cyclolanost-25(251)-ene] (42) (Figure 2).

### Pharmacological activities of extracts, fractions, and isolated constituents

Several studies have been conducted to identify the active molecules in coconut and their possible pharmacological and biological activities. Various extracts, fractions, and isolated compounds from different parts of the coconut fruit were tested, showing different activities, including antihypertensive; analgesia; vasodilation; protection of kidney, heart, and liver functions; protection against ulcers; and anti-inflammatory, anti-oxidant, anti-osteoporosis, antidiabetes, antineoplastic, bactericidal, antihelminthic, antimalarial, leishmanicidal, antifungal, and antiviral activities (43–47). These effects are described below and also listed in Supplementary Table S1.

### Analgesic activity

Crude husk-fiber extract and two aqueous extract fractions of molecular weights less than (F1) and greater than (F2) 1 kDa were studied for their analgesic activity by acetic acid-induced abdominal writhing, tail-flick, and hot plate tests in mice (44). All three extracts induced peripheral and central antinociceptive activity. Oral administration of the crude extract (50, 100, or 150 mg/kg) significantly inhibited writhing by 24%, 34%, and 52.4%, respectively, when compared with a control group. Fractions F1 and F2 reduced total writhing at 10 and 50 mg/kg. In the tail-flick test, oral pre-treatment with crude extract (100 and 150 mg/kg), F1 (10 and 50 mg/kg), or F2 (10 and 50 mg/kg) produced effects better or similar to morphine (5 mg/kg) until 80 min. However, with the exception of F1 (50 mg/kg, 60 min after administration), neither crude extract (150 mg/kg) nor F2 (50 mg/kg) significantly increased the latency of mice response to thermal stimulation in the hot-plate test. The mechanism of action of the extracts were also evaluated using the opioid antagonist naloxone (5 mg/kg), which inhibited the antinociceptive effect of the crude extract, F1, and F2, indicating a probable action on opioid receptors.

In another study, an ethanol extract of the husk fiber (40, 60, or 80 mg/kg) showed significant analgesic properties, as indicated by a reduction in the number of writhes and stretches induced in mice by 1.2% acetic acid (41). The results were similar to those in animals that received aspirin (68 mg/kg), paracetamol (68 mg/kg), or morphine sulfate (1.15 mg/kg). Furthermore, administration of the ethanol extract along with morphine or pethidine not only produced analgesia in mice but also potentiated the analgesic effect of these two drugs.

These studies were performed using coconut husk fiber extracts, suggesting that this part of the plant is a highly potent analgesic. *Cocos nucifera* may enable the production of new low-cost medicines for several ailments and may provide a very inexpensive source of new analgesic drugs. Further investigations are warranted. Further bioassay-guided fractionation and isolation of specific molecules are highly recommended so that the chemical moiety responsible for the activity can be identified and its mechanism of action established.

### Anti-inflammatory activity

Aqueous crude extracts of husk fiber of *C. nucifera* are used to treat arthritis and other inflammatory ills in Northeastern Brazil's traditional medicine (7).

A study using animal models of inflammation (formalin test and subcutaneous air pouch model) showed that aqueous crude extracts of *C. nucifera*var. *typica* (50, or 100 mg/kg) significantly inhibited (P<0.05) the time that animals spent licking their formalin-injected paws and reduced inflammation induced by subcutaneous carrageenan injection by reducing cell migration, extravasation of protein, and TNF-α production (45)

Husk fiber extracts were also tested on rat paw edema induced by carrageenan, histamine, and serotonin (44). Animals were pre-treated by oral administration of crude extract (50, 100 or 150 mg/kg), F1 or F2 (1, 10, or 50 mg/kg), promethazine (30 mg/kg), or methysergide (5 mg/kg). The crude extract significantly (P<0.05) reduced histamine (at 150 mg/kg) and serotonin-induced rat paw edema (at 100 and 150 mg/kg). Even when mice were treated with 1 mg/kg of F1, a significant inhibitory effect was observed in histamine and serotonin-induced edema. However, F2 did not inhibit the edema induced by any pro-inflammatory agent.

Animal tests revealed significant activity supporting the use of these husk fiber extracts in traditional medicine (35). The chemical constituents responsible for their activity should be isolated, identified, and researched to establish safety doses.

### Anti-bacterial, antifungal, and anti-viral activities

Brushing the teeth with fibrous coconut husks is a common oral hygiene practice among rural people of South India (46). In this context, the antimicrobial properties of alcoholic extracts of the husk against common oral pathogens were analyzed by the agar well diffusion method (47). There was significant concentration-dependent antimicrobial activity, expressed as a zone of inhibition with respect to all tested organisms except *Actinomyces* species. However, the effect of the *C. nucifera* extract was less than that of chlorhexidine.

Ethanolic (cold and hot percolation), dry-distilled, and aqueous extracts of coconut endocarp were compared with gentamicin and ciprofloxacin for their antibacterial activities against methicillin-resistant *Staphylococcus aureus* (MRSA), methicillin-sensitive *S. aureus*, *Pseudomonas aeruginosa*, *Escherichia coli*, *Klebsiella pneumonia*, *Acinetobacter baumannii*, *Citrobacter freundii*, *Enterococcus*, *Streptococcus pyrogens*, *Bacillus subtilis*, and *Micrococcus luteus*using the Kirby-Bauer disc diffusion method. The endocarp extracts showed strong antimicrobial activity against *B. subtilis*, *P. aeruginosa*, *S. aureus*, and *M. luteus* but had no effect on *E. coli* (26). The dry-distilled extract (1 mg/mL and 200 μg/mL) could inhibit the growth of *B. subtilis* and *Aspergillus* spp. but was inactive against *R. oligosporus* at all concentrations (48). The crude aqueous extract of husk fiber and five fractions obtained by thin layer chromatography (TLC) were also tested (10, 50, and 100 mg/kg) against *E. coli*, *S. aureus*, and MRSA via agar diffusion; they were active only against *S. aureus* and MRSA, with a minimum inhibitory concentration (MIC) of 1024 mg/mL for both (45).

In another study, the antimicrobial activity of mesocarp powder extracted with six common organic solvents was evaluated by the disk diffusion method (49). The pathogens *E. coli*and *S. typhi* were used. The antimicrobial activity against *E. coli* was higher with the benzene solvent, while bioactivity toward *S. typhi* was more effective with the diethyl ether extract. Potential bio-components responsible for the antimicrobial activity were identified as tocopherol, alcohol palmitoleyl, cycloartenol, and β-sitosterol.

The *in vitro* antilisterial activities and time kill regimes of crude aqueous and *n*-hexane extracts of the husk fiber of *C. nucifera* were tested (50). The aqueous extracts were active against 29 of 37 *Listeria* isolates examined, while the *n*-hexane extracts were active against 30 (both at 25 mg/mL). The diameters of the zones of inhibition were 12–17 mm and 12–24 mm, respectively, while those of the control antibiotics were 20–50 mm for ampicillin and 22–46 mm for tetracycline. The MICs of the susceptible bacteria were 0.6–2.5 mg/mL for the aqueous fraction and 0.6–5.0 mg/mL for the *n*-hexane extract. The mean reduction in viable cell count in the time kill assay with the aqueous extract ranged from 0.32 to 3.2 log_10_ CFU/mL after 4 h of interaction and from 2.6 to 4.8 log_10_ CFU/mL after 8 h at 1× and 2× MIC. With the *n*-hexane extract, the values were 2.8–4.8 log_10_CFU/mL after 4 h of interaction and 3.5–6.2 log_10_ CFU/mL after 8 h in 1× and 2× MIC. For the aqueous extract, bactericidal activity was observed against three of the tested *Listeria* strains at a concentration of 2× MIC after 8 h exposure, while the *n*-hexane fraction was bactericidal against all five test bacteria at both MICs after 8 h.

In studies with crude extract and five TLC fractions (I-V) of fiber mesocarp of *C. nucifera* fruit, *in vitro* antimicrobial activity was seen in all trial strains of *S. aureus* tested with fractions II-V (7). Antifungal activity was demonstrated as growth inhibition of *Candida albicans*, *Cryptococcus neoformans* or *Fonsecaea pedrosoi*. Antiviral action was only seen with the crude extract and fraction II. The antifungal, antimicrobial, and antiviral effects were attributed to condensed tannins and catechins present in the crude extract and fractions II-V, especially fraction II, which had a higher concentration of these compounds.

Studies with alcohol extract of ripe dried coconut shell have demonstrated action against *Microsporum canis*, *M. gypseum*, *M. audouinii*, *Trichophyton mentagrophytes*, *T. rubrum*, *T. tonsurans*, and *T. violaceum* (51). This activity was attributed mainly to the high content of phenolic compounds. In another study, virgin oil from coconut pulp prevented growth of *C. albicans* (52).

Coconut oil is very effective against a variety of viruses with lipid capsules, such as visna virus, cytomegalovirus, and Epstein-Barr virus (53). The medium chain saturated fatty acids from coconut oil destroy and break the membranes and interfere with viral maturation.

These reports indicate that various parts of *C. nucifera* should be further tested for antibacterial, antifungal, and antiviral activities in different animal models. Future studies should consider formulations and exact dose levels suitable for use in humans to treat various strains of bacteria, viruses, and fungi.

### Antioxidant activity

There is considerable interest in the consumption of certain foods to prevent the onset of diseases. Evidence suggests that diets rich in phenolic compounds can significantly enhance human health because of the effects of phenolic antioxidants (54). Studies with virgin coconut oil (VCO) indicated that the total phenolic content was almost seven times that of commercial coconut oil, because the process of obtaining refined oil destroys some of the biologically active components (55). In the 1,1-diphenyl-2-picrylhydrazyl (DPPH) test, VCO had higher antioxidant activity compared to refined coconut oil (56).

The antioxidant activity of *C. nucifera* endocarp extracts was evaluated by DPPH radical scavenging, nitric oxide radical scavenging, and alkaline dimethyl sulfoxide (DMSO) methods. The DPPH analysis demonstrated that ethanolic (cold and hot percolation), dry-distilled, and aqueous extracts of endocarp had significant antioxidant activity (4.1828, 3.31, 20.83, 1.0179 μg/mL, respectively) comparable with that of standard ascorbic acid (48).

In another study, the antioxidant potential of four varieties of coconut (green dwarf, yellow dwarf, red dwarf, and Malaysian yellow) were evaluated and compared with industrialized and lyophilized water of the green dwarf variety (57). All varieties were effective at eliminating DPPH (50% inhibition concentration (IC_50_) 73 mL) and nitric oxide (0.1 mL; inhibition percent (IP) 29.9%) as well as the *in vitro* production of thiobarbituric acid (1 mL; IP 34.4%). The green dwarf variety, which is commonly used, was especially potent compared with another variety of coconut. In cell culture, green dwarf water protected against oxidative damage induced by hydrogen peroxide.

Micronutrients, such as inorganic ions and vitamins present in coconut water, play vital roles in helping the antioxidant defense system of the human body (58). Some evidence points toward an antioxidant action of coconut water. Thus, administering coconut water (6 mL/100 g of body weight) to female rats intoxicated with carbon tetrachloride recovered the action of antioxidant enzymes (superoxide dismutase and catalase levels) and decreased lipid peroxidation (59). Coconut water is also rich in L-arginine (30 mg/dL), which significantly reduces the generation of free radicals (60) and has antioxidant activity (61), as well as ascorbic acid (15 mg/100 mL), which decreases lipid peroxidation in rats (62).

In summary, many parts of *C. nucifera* plants have proven to contain phenolic compounds and flavonoids that support antioxidant activity.

### Antineoplastic activity

Different molecular weight fractions of husk fiber aqueous extracts of *C. nucifera* (typical A variety, commonly known as “olho-de-cravo”, and the common variety) were tested on human erythroleukemia cell line K562 and Lucena 1, a multidrug-resistant (MDR) and vincristine-resistant derivative of K562. Both varieties showed cytotoxicity against K562 cells and decreased by 50% the viability and anti-MDR activity of Lucena 1 cells. In both varieties, the antitumoral activity was concentrated in fractions with molecular weights between 1 and 10 kDa (63).

There is great potential for future research on antineoplastic activity, as only one study has been reported. Because coconut is extensively cultivated in Brazil and its fiber is often discarded, it may offer an inexpensive source for new antineoplastic drugs.

### Antiparasitic activity

The antihelminthic activity of liquid extract of the bark of the green coconut (LBGC), as well as butanol extract obtained from LBGC, was tested on mouse intestinal nematodes (28). Thirty-six naturally infected mice were distributed into 6 treatment groups as follows: group I, 1000 mg/kg of LBGC; group II, 2000 mg/kg of LBGC; group III, 500 mg/kg of butanol extract; group IV, 1000 mg/kg of butanol extract; group V, 0.56 mg/kg febendazole; and group VI, 3% dimethylsulfoxide. The LBGC did not show antihelminthic activity against the mouse nematodes compared with the negative control group (P>0.05). However, the butanol extract at 500 and 1000 mg/kg had mean efficacy of 62.72% and 98.36%, respectively (P<0.05).

The ovicidal and larvicidal activity of the liquid from the coconut husk (LCCV) and butanolic LCCV extract were also tested against *Haemonchus contortus* (28). In egg hatching and larval development tests, 2.5 mg/mL LCCV and 10 mg/mL butanolic extract showed 100% ovicidal activity. Their larvicidal effects were 81.30% and 99.80% at 65 and 80 mg/mL, respectively.

These results suggest that coconut extracts can be used to control gastrointestinal nematodes and that more studies are needed to evaluate their use in humans.

### Anti-*Leishmania* activity

The *in vitro* leishmanicidal effects of *C. nucifera* on *Leishmania amazonensis* were evaluated (33). The polyphenolic-rich extract obtained from coconut husk fiber completely inhibited the cellular growth of *L. amazonensis* promastigote forms (MIC 10 μg/mL) and killed 100% of both developmental stages of the parasite after 60 min (at 10 and 20 μg/mL). In addition, pretreatment of mouse peritoneal macrophages with 10 μg/mL of *C. nucifera* polyphenolic-rich extract reduced by approximately 44% their rate of association with *L. amazonensis*promastigotes with a simultaneous increase of 182% in nitric oxide production by macrophages compared with untreated macrophages.

Ethyl acetate extract (EAE) from husk fiber water was tested against *L. braziliensis* infected hamsters (35). Administering EAE (0.2 mL, 300 mg/kg) for 21 consecutive days did not reduce edema of infected footpad nor the weight of lymph node drainage but reduced skin lesions after 14 days.

These results offer new promise for the development of drugs against leishmaniasis from coconut extracts because of their potent effects and the absence of *in vivo* allergic reactions or *in vitro* cytotoxic effects in mammalian systems. Further studies with these and other species of the parasite are necessary to elucidate the role of *C. nucifera* in eliminating this etiological agent and its healing activity.

### Depressant and anticonvulsant activity

Ethanol extract of root of *C. nucifera* (EECN) at 40, 60, and 80 mg/kg, *ip,* significantly enhanced the duration of sleep induced by pentobarbital (40 mg/kg, *ip*), diazepam (3 mg/kg, *ip*), and meprobamate (100 mg/kg, *ip*) in mice, suggesting a probable depressive action on the central nervous system (41). The anticonvulsant action of EECN was also observed in pentylenetetrazole-induced seizure models. In the animals treated with 25 mg/kg, *ip*, EECN, 60.7% had seizures and died 30 min later. In the group that received EECN at 80 mg/kg, *ip*, no animals had seizures or died, even after 24 h. The components responsible for this depressant activity need to be identified, as well as the mechanism involved in this action. Research on the toxicity of these extracts is also warranted to guarantee the safety of possible future treatments.

### Renal protective activity

Coconut water had prophylactic action against nephrolithiasis in an experiment with a Wistar rat model (64). Rats were divided into three groups. Group I (control) was fed standard rat diet. Group II was administered 0.75% ethylene glycol in drinking water to induce nephrolithiasis. Group III was given coconut water in addition to ethylene glycol. All treatments lasted 7 weeks. Analysis of urine samples revealed a drastic decrease in the number of calcium oxalate crystals in group III compared with group II. Coconut water also significantly lowered the levels of creatinine and urea in group III animals, significantly reduced lipid peroxidation (group II: 38.99±3.36 mol MDA/mg protein/15 min; group III: 27.68±2.45 mol MDA/mg protein/15 min) and decreased the enzyme activities of superoxide dismutase and catalase. These results demonstrate that coconut water has important properties against urolithiasis and therefore must be investigated as a potential treatment for this condition.

### Antimalarial activity

Antimalarial activity of different crude methanol extracts (50, 100, 200, and 400 mg/kg, treated orally) was investigated *in vivo* against *Plasmodium berghei* (NK65) in mice during early, established, and residual infections. Chloroquine (20 mg/kg) and pyrimethamine (1.2 mg/kg) were used as reference drugs. The methanol white flesh extract of *C. nucifera* produced a dose-dependent chemotherapeutic activity in all three *in vivo*-assessment models. In the established malaria infection, there was a significant (P<0.05) decrease after treatment with the extract (200 and 400 mg/kg) compared to the reference drug for the treatment of the disease (25). These results suggest that the Malaysian folkloric medicinal application of *C. nucifera* has a pharmacological basis; however, chloroquine was much more effective at suppression and curing, and the extract did not increase the survival time of infected mice, indicating the need for additional studies to elucidate how *C. nucifera* can be used to treat malaria.

In another study, the antimalarial and toxicity potentials of husk fiber extracts of five Nigerian varieties of *C. nucifera* were evaluated *in vitro*. The results showed that only the West African Tall ethyl acetate extract fraction (WATEAEF) was active against *P. falciparum* W2 strain with a selectivity index of 30.3. The phytochemicals present in the WATEAEF were alkaloids, tannins, and flavonoids. The same extract fraction was active *in vivo* against *P. berghei* NK65, causing more than 50% reduction in parasitemia on days 4 and 6 after inoculation at various doses. However, parasitemia varied on days 8 and 10, and results with WATEAEF were no better than with chloroquine. Additionally, treatment with 250 and 500 mg/kg body weight WATEAEF significantly increased (P<0.05) plasma creatinine concentration compared with controls (65). Despite the reduction in parasitemia, the extract cannot yet be considered an appropriate treatment for malaria. More studies are needed to clarify the adverse effects and effectiveness of the coconut extracts, especially in infections caused by *P. falciparum* (the main agent in humans).

### Antitrichomonal activity

The crude methanol extracts of 22 plants used in Mexican folk medicine were tested *in vitro* against *Trichomonas vaginalis*. The susceptibility tests were performed using a previously described subculture method (66). Trophozoites (4×10^4^) were incubated for 48 h at 37°C in the presence of different concentrations (2.5–200 mg/mL) of the crude extracts in DMSO. Each test included metronidazole as a positive control, a negative control (culture medium plus trophozoites and DMSO), and a blank (culture medium). The experiments were performed in duplicate and repeated at least three times. The crude methanol extract of *C. nucifera* husk fiber demonstrated excellent antitrichomonal activity (IC_50_ value of 5.8 μg/mL), standing out among the other tested plants, although the activity was less than that of metronidazole. Further research is needed to isolate the substances responsible for this activity and to test appropriate doses so that they can be used in the treatment of trichomoniasis (24).

### Cardioprotective activity

An important biological action of coconut was demonstrated using an experimental model of myocardial infarction induced by isoproterenol in rats (67). Feeding these animals with tender coconut water (West Coast Tall variety) protected against the induction of myocardial infarction and decreased mitochondrial lipid peroxidation.

In another study, dietary coconut sprout (West Coast Tall variety) was tested on isoproterenol-induced myocardial infarction in rats (68). There was a decrease in the levels of cardiac markers (CK-MB and troponin-T) in serum of the group pretreated with coconut sprout (50, 100, or 200 mg/100 g body weight) orally for 45 days. Rats fed with 100 mg/100 g body weight showed better results than other treatment groups. In addition, pretreatment with coconut sprout decreased oxidative stress in the heart and increased antioxidant status. Biochemical analyses showed that sprouts contains bioactive components, such as vitamins, alkaloids, and polyphenols.

Tender coconut water could also reduce total cholesterol, very-low density lipoprotein, low density lipoprotein, and triglyceride levels in serum (69). Administering coconut water (4 mL/100 g body weight) in male rats counteracted the increases in these substances promoted by cholesterol feeding.

The results presented here support the cardioprotective effects of coconut water. Its administration could reduce oxidative stress and cell damage in animals with induced myocardial infarction and reverse increases in cholesterol levels in animals fed high-fat diets. Therefore, further research is warranted on its potential use to prevent a second ischemic event or in the treatment of dyslipidemic states.

### Hepatoprotective activity

The hepatoprotective effect of tender coconut water was investigated in carbon tetrachloride (CCl_4_)-intoxicated female rats. The animals were divided into three groups: normal control rats, CCl_4_-treated control rats, and tender coconut water pretreated rats intoxicated with CCl_4_. Carbon tetrachloride caused elevated serum glutamate oxaloacetate transaminase and glutamate pyruvate transaminase levels and also lead to liver necrosis and fatty liver, while rats pretreated with coconut water showed decreased activities of these enzymes (59). With only one report describing these effects, there remains room to develop studies to define the real role of *C. nucifera* in this action.

### Antidiabetic activity

The antidiabetic activity of purified coconut kernel protein (CPK) was evaluated in alloxan-induced diabetes (150 mg/kg body weight, *ip*) (70). CPK was isolated from dried coconut kernel and administered to Sprague-Dawley rats with a semi-synthetic diet for 45 days. It attenuated the increase in glucose and insulin levels in these diabetic rats. Glycogen levels in the liver and the activities of carbohydrate-metabolizing enzymes in the serum of treated diabetic rats reverted to normal levels compared with healthy control animals. Histopathology revealed that CPK feeding also reduced the diabetes-related pancreatic damage in treated rats compared with the diabetic control. These results are probably due to the effects of CPK on pancreatic β cell regeneration through arginine, an important amino acid found at high concentrations in CPK by HPLC.

The effects of mature coconut water were also evaluated and compared with glibenclamide in alloxan-induced diabetic rats (71). Treatment of diabetic rats with lyophilized mature coconut water (1000 mg/kg body weight) or glibenclamide (0.6 mg/kg body weight) reduced blood glucose levels (129.23±1.95 and 120±2.3 mg/dL, respectively) when compared with the untreated control (275.32±4.25 mg/dL). Coconut water also increased insulin levels and liver glycogen concentrations and reduced glycated hemoglobin levels in diabetic rats. In addition, elevated levels of liver function enzymes markers like alkaline phosphatase, serum glutamate oxaloacetate transaminase, and serum glutamate pyruvate transaminase in diabetic rats were significantly reduced upon treatment with mature coconut water. It was also observed that diabetic rats showed altered levels of blood urea, serum creatinine, and albumin, and the albumin/globulin ratio was significantly reversed by treatment with mature coconut water and glibenclamide.

Administering immature coconut inflorescence methanol extract (West Coast Tall variety) to diabetic rats significantly reduced fasting glucose levels and increased insulin levels compared with a diabetic control (72). The 200 mg/kg body weight dose showed better antihyperglycemic effects than other doses.

These studies demonstrated that different parts of *C. nucifera*can benefit diabetic rats, similar to oral hypoglycemic agents currently used clinically to control diabetes. Therefore, clinical trials and biochemical analyzes are recommended to isolate the compounds responsible for these actions and to establish them as drugs.

### Effects on bone structure

VCO was investigated in postmenopausal osteoporosis rats to determine its effects on bone microarchitecture (73). Rats were supplemented with VCO (8% mixed with the standard rat chow diet) for 6 weeks. VCO administration significantly increased bone volume, prevented a reduction in trabecular number, and lowered the trabecular separation compared with the ovariectomized control. Bone histology revealed that the trabecular bones of the ovariectomized group appeared to be sparser and less dense than in the group treated with VCO. Treatment of ovariectomized rats with VCO seemed to reverse the effects of estrogen deficiency on bone structure. With only one report on the anti-osteoporosis activity of *C. nucifera* available, there is a need for further studies.

### Antihypertensive activity

The anti-hypertensive activity of an ethanolic extract of *C. nucifera* endocarp (EEC) using the deoxycorticosterone acetate (DOCA) salt-induced model of hypertension was observed. Administering EEC significantly reduced the mean systolic blood pressure in DOCA salt-induced hypertensive rats (from 185.3±4.7 to 145.6±6.1 mm Hg). This effect was attributed to the direct activation of the nitric oxide/guanylate cyclase pathway as well as stimulation of muscarinic receptors and/or the cyclooxygenase pathway. These activities can be explained by the presence of phenolic compounds and flavonoids in the extract used (74). Based on these results, *C. nucifera* should be studied further for its potential use against cardiovascular diseases.

## Toxicity

Several studies have investigated the toxicological properties of *C. nucifera.* One paper verified the effect of ethyl acetate extract of *C. nucifera* fiber on physiological parameters and on topical inflammation induced by xylene in animal models (75). Regarding the physiological parameters and macroscopic aspects of the lymphoid organs in this study, neither mortality nor any symptom of toxicity was observed in the animals.

The possible toxic effects of crude extract, F1, and F2 (see above) of *C. nucifera* mesocarp were evaluated in mice (44). The oral treatment of mice over 5 days with a single dose (500 mg/kg) caused no behavioral changes. No injury or bleeding stomachs were observed.

Another study evaluated the toxicity of a methanol extract of *C. nucifera* endocarp (25). Five female and five male mice received a single dose orally (5000 mg/kg) of this extract. All male and female rats were observed for signs of toxicity and mortality on the day of dosing; at 1, 3, and 4 h after administration; and then twice daily for 14 days. No signs of toxicity and mortality were recorded, and all animals gained weight during the observation period.

Acute, subchronic, and chronic toxicity from liquid mesocarp of green coconut (LMGC) and butanol extract obtained from the LMGC were tested in mice and rats (28). No acute lethal effects were observed in mice receiving a dose of 3000 mg/kg orally of either extract. In contrast, when LMGC and butanol extract were administered intraperitoneally at doses of 500 and 700 mg/kg, no animal survived. In subchronic toxicity tests, the rats treated with LBGC had significantly higher white blood cell, neutrophil, red blood cell, hematocrit, and platelet counts. In the chronic toxicity test, the group treated with LBGC showed higher values for neutrophils, white blood cells, basophils, and platelets (P<0.05). However, in the subchronic and chronic toxicity tests, no hematological parameters differed significantly in the group treated with butanol extract (P>0.05). Only triglycerides were higher (P<0.05) in the group treated with LBGC during the chronic toxicity test. Rats treated with both extracts had no histopathological changes related to toxicity, nor did weight gain differ between treated and control groups (P>0.05). In conclusion, both extracts showed low toxicity for those parameters.

## Conclusion

*Cocos nucifera* is a widely dispersed plant that has important pharmacological effects with low toxicity. Furthermore, medicinal use of *C. nucifera* has an environmental appeal, since this plant is widely used in the food industry and use of discarded plant parts will reduce waste and pollution. The pharmacological effects of the plant differ according to the part of the plant or fruit used. Antioxidant activity predominated in the constituents of the endocarp and coconut water. In addition, the fiber showed antibacterial, antiparasitic, and anti-inflammatory activities. Only the ethanolic extract of the root had depressant and anticonvulsant action on the central nervous system. Coconut water seems to have protective effects, e.g., on the kidney and heart, and antioxidant activity, as well as a hypoglycemic effect.

Some limitations of the studies on *C. nucifera* must be acknowledged. First, the studies have focused on the effects of different parts of the plant but without demonstrating the mechanisms underlying these actions. Second, formulations based on parts of the plant must be developed to conduct clinical trials.

Considering the diversity of pharmacological properties, future research into *C. nucifera* should be encouraged. The main goals should be to isolate specific compounds, to clarify the mechanisms involved in the pharmacological effects, and to investigate possible toxic effects to produce safe phytotherapies.

Several factors may limit such studies. Geographical and seasonal variations among countries and regions can influence the chemical composition of the studied material. Therefore, standardized procedures for collecting samples and quantifying compounds should be used to assure the reproducibility of results.

## Supplementary Material


