# Supplementary material for: Cocos nucifera (L.) (Arecaceae): A phytochemical and pharmacological review
Source: Braz J Med Biol Res. 2015 Aug 18;48(11):953–64. doi: 10.1590/1414-431X20154773 (PMC4671521; doi:10.1590/1414-431X20154773)
Supplement: Supplementary file 1 [file 1414-431X-bjmbr-48-11-00953-supp4773.pdf]

Table S1. Pharmacological Activities of various extracts of *Cocos nucifera* (L) and its fractions.

Plant part	Type of extract/fraction	Study model/Tests	Doses	Pharmacological activity	Ethnopharmacological claim	References
Husk fiber	Crude extract (CE); fractions (F1 e F2)	Acetic acid-induced abdominal writhing (*in vivo*). Formalin, hot plate and tail-flick tests	50, 100 or 150 mg/kg, *po*	Analgesic	Use of husk fiber tea for pain.	44
Husk fiber	Crude aqueous extract (CE)	Formalin-induced licking and subcutaneous air pouch (*in vivo*).	10, 50 or 100mg/kg, *po*	Anti-inflammatory	Use of husk fiber tea for arthritis, diarrhea.	45
Husk fiber	Crude extract (CE) and fractions (F1 e F2)	Rat paw edema test.	AE (50, 100,150 mg/kg), *po*<br>F1: 1, 10 50 mg/kg), *po*<br>F2: (1, 10, 50 mg/kg), *po*	Anti-inflammatory		44
Husk fiber	Liquid extracted from the bark of the green coconut (LBGC) and butanolic extract (BE)	Antihelminthic activity on mouse intestinal nematodes	LBGC (1000-2000 mg/kg), *po*<br>BE (500-1000 mg/kg), *po*	Antihelminthic		28
Husk fiber	Crude extract (CE) and fractions (FI-FV)	Reduction of virus titers using TCID50 determinations (*in vitro*).	500 mg/mL	Antiviral		7
Husk fiber	Crude methanol extract	Trichomonas vaginalis trophozoites were incubated in the presence of the crude extracts in dimethyl sulfoxide (DMSO).	2.5-200 µg /mL	Antitrichomonal	Treatment of disorders of urogenital tract associated with infection by *T. vaginalis*.	24
Husk fiber	Crude extract (CE) and fractions (FI-FV)	The agar diffusion method (*in vitro*).	500 mg/mL	Antimicrobial		7
Husk fiber	Crude aqueous extract (CE)	The agar diffusion method (*in vitro*).	10, 50 or 100 mg/kg	Antimicrobial		45
Husk fiber	Aqueous extract (AE) and extract obtained with n-hexane (EnH)	Microorganism culture on agar;	25 mg/mL	Bacteriostatic or bactericidal		50
Husk fiber	Aqueous extract fractions: A, B and C.	Cytotoxicity against leukemic cells (MTT test).	0, 5, 50 or 500 µg/mL.	Antileukemic		63
Husk fiber	Aqueous extract rich in polyphenols (AEP)	*In vivo:* culture of parasites promastigotes of *L. amazonensis. In vitro L. amazonensis* promastigotas was incubated at 26°C for 120 h.	10 and 20 µg/mL of extract, not informed route of administration	Leishmanicidal		33
Husk fiber	Ethyl acetate extract (EAE).	Promastigotes of *L. braziliensis*were inoculated in the right hind paw of hamsters. Paw edema test, the skin lesions and leukocyte parameters.	300 mg/kg, *po*	Leishmanicidal		35
Endocarp	Ethanol extract (RNM-1; RNM-2) Oily liquid obtained from the dry distillation (RNDS).	DPPH test. Nitric oxide radical scavenging. Alkaline DMSO method. Determination of total phenolic compounds, total flavonoids and tannins.	Not informed doses or route of administration	Antioxidant		48
Endocarp	Ethanolic extracts, dry distilled extract and aqueous extract	Agar diffusion test was performed to evaluate antibacterial activity against *S. aureus*, *P. aeruginosa*, *K. pneumonia*, *E. coli*, *A. bauminii*, *Citrobacter freundii*, Enterococcus, *S. pyrogens*, *Bacillus subtilis* and *Micrococcus luteus*.	Not informed doses or route of administration	Antimicrobial activity of the endocarp extracts shows strong activity against *B. subtilis*, *P. aeruginosa*, *S. aureus*, *M. luteus*. Value of MIC was found between 300-350 µg/mL against *B. subtilis*.		48
Endocarp	Ethanolic extract (EE)	*In vitro*: Aortic rings with and without endothelium.<br>*In vivo*: Model of hypertension in uninephrectomized male rats with	*In vitro:* 0.25-2 mg/mL<br>*In vivo:* 300 mg/kg, *ip*	Antihypertensive	The fruit of *Cocos nucifera* L. has long been used in folk medicine for the treatment of cardio-	74

		induced salt.			metabolic diseases.	
Mesocarp	Mesocarp extract (MS)	Agar diffusion test	Not informed doses or route of administration	Antimicrobial	In the Indian subcontinent, is used as hydration therapy for cholera, diarrhea and dysentery; addition to the treatment of cancer.	49
Coconut water	Not applicable	Liver injury was induced by CCl4. Tests and measurements: Liver enzymes and oxidative stress	6 mL/100 g of body weight. Not informed route of administration	Antioxidant	Used for relief of fever, intestinal disorder. Oral rehydration.	59
Coconut water	Virgin coconut oil (VCO)	Ovariectomized rats	VCO 8% added to the regimen. Not informed route of administration	Anti-osteoporosis		73
Coconut water of four varieties	Not applicable	Cell culture of lung fibroblasts to study the effect of caffeic acid on oxidative stress. DPPH assay, scavenging of nitric oxide, TBARS measurement.	Not informed doses or route of administration	Antioxidant		57
Coconut water	Not applicable	Nephrolithiasis model Wistar rats. Determination of lipid peroxidation, SOD and catalase. Chemical analysis of 24 h urine. Analysis of renal function serum sample. Isolation of total RNA.	Not informed doses or route of administration	Nephroprotective		64
Mature coconut water (MCW)	Not applicable	Alloxan-induced diabetes model in rats. Glucose, insulin and glycated hemoglobin were estimated. Blood urea was calculated. Concentration of urinary nitrate, serum proteins was calculated albumin, TGO and TGP was estimated. Serum creatinine and nitric oxide synthase (NOS) activity were estimated.	4 mL/100 g, intragastric.	Reverses the increase in the concentration of urea, creatinine and serum nitrite. Animals receiving MCW + glibenclamide showed increased NOS activity in the liver, as well as increased plasma concentration of L-arginine.		71
Albumen solid	Crude methanol extract (CME)	Animal model of malaria	50, 100, 200 and 400 mg/kg, *po*	Antiparasitic	Treat malaria, fever, taeniasis, schistosomiasis and ancylostomiasis.	25
Protein of albumen solid coconut (CAP)	Not applicable	Hypercholesterolemic rats	80 g CAP/kg diet. Not informed route of administration	Hypolipidemic and antiperoxidative		61
Fresh roots	Ethanol extract of *C. nucifera* (EECN)	Acetic acid-induced abdominal writhing. Hot plate test.	40, 60 or 80 mg/kg, *ip*	Analgesic		41
Fresh roots	Ethanol extract of *C. nucifera* (EECN)	Pentylenetetrazole-induced seizures model	25-80 mg/kg, *ip*	Anticonvulsant		41
Fresh roots	Ethanol extract of *C. nucifera* (EECN)	Test induced sleep pentobarbital, meprobamate and diazepam in mice.	40, 60 or 80 mg/kg, *ip*	Potentiation of pentobarbital-induced sleep.		41

MIC: minimum inhibitory concentration; DMSO: dimethyl sulfoxide; NOS: nitric oxide wynthase; *ip*: intraperitoneal route; *po*: oral route.
